# A Spatial Adaptive Algorithm Framework for Building Pattern Recognition Using Graph Convolutional Networks

**DOI:** 10.3390/s19245518

**Published:** 2019-12-13

**Authors:** Weijia Bei, Mingqiang Guo, Ying Huang

**Affiliations:** 1 School of Geography and Information Engineering, China University of Geosciences (Wuhan), Wuhan 430074, China; 20171000718@cug.edu.cn; 2 Wuhan Zondy Cyber Technology Ltd. Co., Wuhan 430074, China; huangying@mapgis.com

**Keywords:** building pattern, node classification, graph partition, graph convolutional networks, random forest, graph convolutional neural networks, machine learning

## Abstract

Graph learning methods, especially graph convolutional networks, have been investigated for their potential applicability in many fields of study based on topological data. Their topological data processing capabilities have proven to be powerful. However, the relationships among separate entities include not only topological adjacency, but also correlation in vision, for example, the spatial vector data of buildings. In this study, we propose a spatial adaptive algorithm framework with a data-driven design to accomplish building group division and building group pattern recognition tasks, which is not sensitive to the difference in the spatial distribution of the buildings in various geographical regions. In addition, the algorithm framework has a multi-stage design, and processes the building group data from whole to parts, since the objective is closely related to multi-object detection on topological data. By using the graph convolution method and a deep neural network (DNN), the multitask model in this study can learn human thoughts through supervised training, and the whole process only depends upon the descriptive vector data of buildings without any ancillary data for building group partition. Experiments confirmed that the method for expressing buildings and the effect of the algorithm framework proposed are satisfactory. In summary, using deep learning methods to complete the tasks of building group division and building group pattern recognition is potentially effective, and the algorithm framework is worth further research.

## 1. Introduction

Buildings are important entities in the fields of city computing and city perception. The distributive characteristics of different building groups can be visually summarized as patterns considered as the fine-grained features of the city. In addition, patterns of building groups play an important role in map generalization and navigation [1,2], and the indices (e.g., the area, perimeter, orientation and compactness) of buildings are descriptive enough for deep learning methods to accomplish some classical tasks of building pattern classification [3]. In general, building patterns can be divided into regular patterns and irregular patterns. Grid-like patterns are the main manifestation of regular patterns, while irregular patterns mainly consist of I-shape, L-shape and independent types [4].

Existing methods for building pattern recognition usually partition building groups with the help of road networks and other ancillary data [5,6,7], and thus the applicability in some geographic analysis scenarios is weakened [8]. Therefore, proposing a building group partition method with high accuracy is essential for the data independence of finishing the building pattern recognition task.

The building pattern recognition task is equivalent to the multi-object recognition task, and the difference is that the former is based on topological data, compared to the traditional multi-object recognition task of computer vision. The building group partition operation in the building pattern recognition process is like region proposal methods. Fast R-CNN [9] and its derivative algorithms are a kind of effective, multi-object recognition framework [10,11]. The contour of the target object is often irregular. Hence convolutional neural networks (CNNs) are used to calculate the probability of whether the proposal region contains target objects [12]. On the basis of Fast R-CNN, the appearance of Mask R-CNN brought the research to the stage of instance segmentation [13]. Mask R-CNN is also based on region proposal methods and identifying each pixel as the background or as a part of the object by a fully convolutional network (FCN) [14]. To identify all building patterns in a building group, the instance segmentation idea is transferable. Inspired by the usage of the FCN in Mask R-CNN, in this study we summarize three spatial states of one building: the edge state, the inner state and the free state. Specifically, there is an analogy between the buildings of the edge state and the contours of an object output by Mask R-CNN, and the method proposed in this study classifies each building into one of the three states mentioned above by using the graph convolutional network (GCN) model, like the usage of the FCN.

Graph neural networks (GNNs) and GCNs exhibit excellent performance based on topological data in different research fields, including social networks [15], protein interface prediction [16], disease prediction [17,18] and remote sensing image processing [19]. GNNs and GCNs accomplish information aggregation according to the adjacent relations between nodes in a graph, aiming to perceive the topological features of different nodes. This notion is closely related to spatial association [20] and the first law of geography [21] because of the natural formation of adjacent relations based on distances. However, in general circumstances, the relationships between objects not only include topological adjacency but also shape similarity, especially in research on building pattern recognition [3,22]. The graph learning methods mentioned above are normally applied to non-Euclidean data, but seldom focus on the visual characteristics and the spatial distribution of the nodes [23].

To perceive the relationships among the graph node and its neighbors, graph embedding is employed, which learns to represent graph nodes with n-dimensional vectors. Graph embedding has a close connection with methods of the representation-based classification (e.g., some up-to-date works, such as LMRKNN [24], TPCRC [25], the novel DCRC method via l2 regularizations [26] and MKFLE [27]). Inspired by the representation learning, graph embedding methods on graph domain (e.g., DeepWalk [28], node2vec [29], LINE [30] and SDNE [31]) and some specific methods like those seen in [32] and [15] are proposed, which accomplish information aggregation based on the adjacent relationships among nodes on graphs. The similarities and the discriminations of the graph nodes are reflected by the representation vectors, with the expectations that the nodes can be correctly classified into their own classes.

In this work, we propose a representation method, named as the shifting degree of adjacency weight, to describe the spatial correlation between the buildings and the visual characteristics of the building nodes. To avoid the overfitting problem [33], the method is rotate-invariant and shift-invariant. Besides, in order to achieve the objective of building pattern recognition, an algorithm framework is proposed in this study. The framework has a multi-stage design and processes the building group data from whole to parts, since the building pattern extraction during the workflow is associated with the multi-object detection on graphs. Additionally, the mentioned graph learning methods encode the nodes by the information passing through and aggregation between adjacent nodes to derive the features of nodes, which solves the problems of the unfixed size of vertices and the uncertain adjacent relationships. In this study, a novel graph convolution operation is introduced for better performance of feature encoding.

With a symmetric normalized Laplacian matrix, the adjacency information of nodes has been normalized during the aggregation operation to address overfitting [15]. In addition, local weight sharing is generalized to graph structure through a polynomial approximation of the Laplacian matrix [32,34]. In addition, a graph convolutional neural network (GCNN) [35,36] is constructed by combining the graph convolution operation with a deep neural network for the graph representation learning related to the building pattern recognition task. Generally, the whole process only depends upon the descriptive vector data of buildings without any ancillary data for building group partition, which improves the applicability of the proposed method.

This study focuses on developing an algorithm framework to finish building group partition and pattern recognition (e.g., I-shaped, L-shaped, grid-like and single type pattern) tasks based only on the vector data of building contours. In addition, experiments for exploring reasonable model structures have been conducted for a satisfactory and convincing result.

The remainder of the paper is structured as follows: Section 2 introduces the experimental datasets for the model training and testing. Section 3 describes the principles of the proposed methods in details, and Section 4 articulates the procedure of the algorithm framework. The experiments and results are presented in Section 5, and some issues are discussed in Section 6. Finally, Section 7 summarizes this research and presents the future works.

## 2. Study Materials

**Figure 1:**
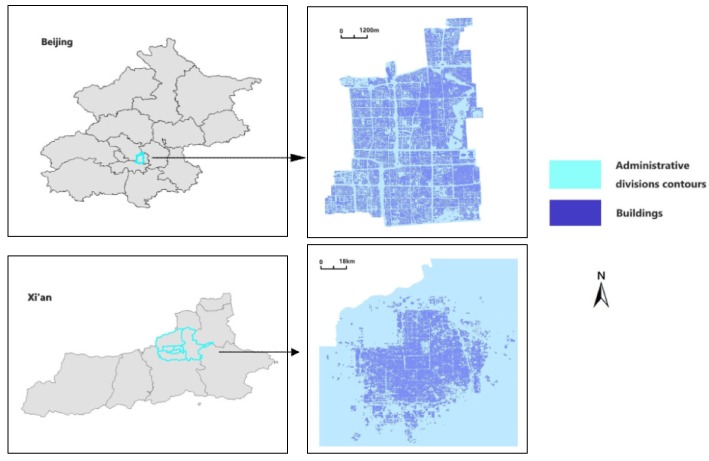
Experimental datasets.

Beijing’s Xicheng District and the core areas of the city of Xi’an (Figure 1) were selected as the study regions. The selection is reasonable because of their long history and their important development positions. The two study regions include various stages of urban development in China, and therefore the building distributions and contours are in accordance with the multiplicity principle. The experimental datasets contain the vector data of building contours at a scale of 1:2000 in the two mentioned areas for 2017. The vector data of each building contour consists of a series of key points recording longitude and latitude data. In the annotation process, we first selected the data of building contours in random rectangular areas as separate data blocks, with 75–154 buildings per block. The models used by the proposed algorithm framework are trained with supervised or semi-supervised learning, and the datasets for the training and testing of different models are labeled according to the specific tasks. In general, three datasets were constructed for the three tasks: building state identification, building node clustering and building pattern recognition.

## 3. Methodology

On the basis of the adjacency relationships among the building nodes, each data block, which is referring to a building group, is processed from whole to parts in the proposed framework with a multi-stage design. All the building nodes are firstly classified into three spatial states for further processes. The four major parts, building node state identification, building group partition, fine-grained partition for building blocks and building pattern recognition, are explained with details in the following sections.

### 3.1. Building Node State Identification

#### 3.1.1. Definition of Three Building Node States

**Figure 2:**
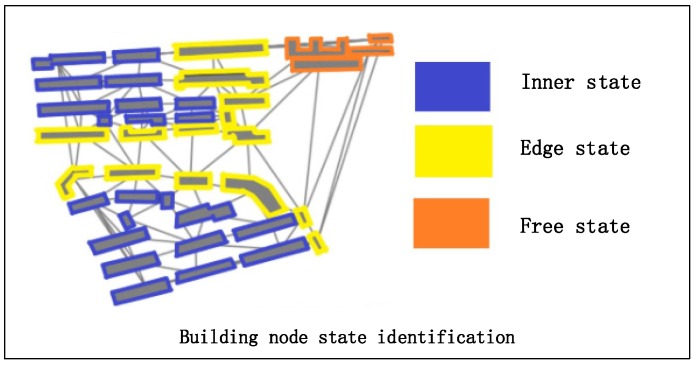
Definition of three building node states.

According to the possible spatial correlation between the building nodes, three kinds of building node states are defined:

- Edge state. Intuitively, the edge state buildings are located on the edge of a building block. Their unique characteristic is that the contrast between the buildings of their two sides is strong (e.g., the bright yellow buildings shown in Figure 2). The contrast embodied by the difference of the descriptive vectors of building nodes will lead to unique feature encoding through the graph convolution operation. Therefore, the definition of edge state is reasonable, and it is indicative of the inner state building.
- Inner state. Buildings located in the same building pattern are similar in terms of size, outline and spatial position; hence, buildings located in the same pattern are defined as the inner state buildings (e.g., the blue buildings shown in Figure 2).
- Free state. Normally, there is one building that exhibits independence because of its spatial distance among others. We define such buildings as free state buildings (e.g., the orange buildings shown in Figure 2).

#### 3.1.2. Descriptive Methods for Building Features

We can quantify the differences stated in Section 3.1.2 through descriptive vectors constructed by the variables summarized in Table 1. The definitions of shifting degree of adjacency weight and orientation are in Section 3.1.2.1 and Section 3.1.2.2, respectively.

##### 3.1.2.1. Definition of the Shifting Degree of Adjacency Weight

**Figure 3:**
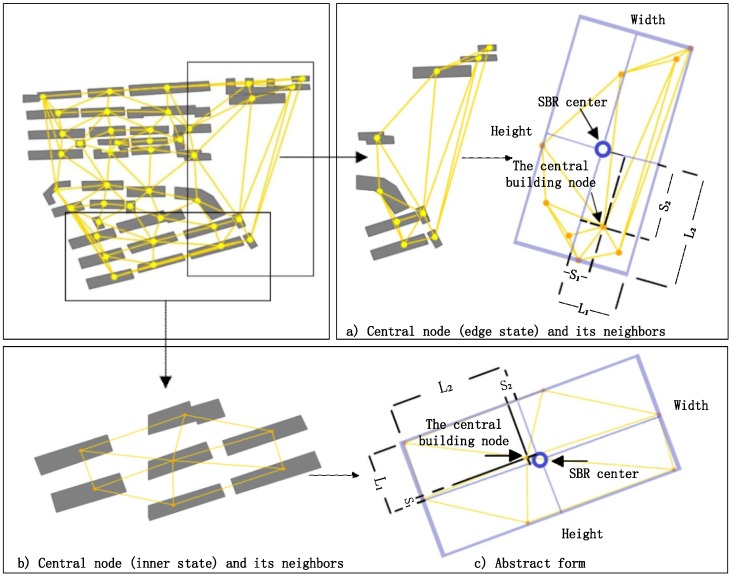
Descriptions of shifting degree of x–y-dimension weight. (**a**) Central node (edge state) and its neighbors. (**b**) Central node (inner state) and its neighbors. (**c**) Abstract form.

The spatial distribution of buildings is fundamental for building group partition. In a building group, it is intuitive to treat buildings that are close to each other as one building block. The sparse part reflects the boundary between two separate partitions. Therefore, the distance between buildings can be used to describe the sparse part [4]. However, distance is merely one descriptive parameter in one-dimensional space, and it is not enough to describe the spatial distribution of the buildings. Thus, we need two-dimensional indices to express the sparsity or the density of the buildings in two-dimensional space (see Figure 3a,c), and the shifting degree of adjacency weight is defined in this study.

As shown in Figure 3a, we first calculate the center coordinate, width and length of the smallest bounding rectangle (SBR) of the central building node and its neighbors. Specifically, (see Figure 3a,c), the width is the edge of the SBR that is parallel to the X axis with counterclockwise rotation of the smallest degree. The adjacent edge is the height of the SBR. The two-dimensional indices are given by

(1) $$D_{w}=\frac{2S_{1}}{L_{1}}$$

(2) $$D_{h}=\frac{2S_{2}}{L_{2}}$$

where $D_{w}$ denotes the shifting degree of adjacency weight in the width direction and $D_{h}$ denotes the shifting degree of adjacency weight in the height direction. The geometrical meanings of $S_{1}$, $S_{2}$, $L_{1}$, and $L_{2}$ are shown in Figure 3a,c where $L_{1}$ denotes the half of the width and $L_{2}$ denotes the half of the height. $S_{1}$ and $S_{2}$ mean the offset distances between the central building node and the center coordinate of the SBR in the width direction and height direction, respectively.

##### 3.1.2.2. Description for Building Orientation

**Figure 4:**
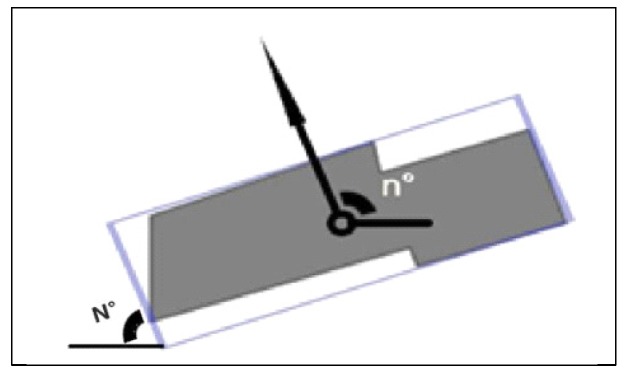
Description of the building orientation.

The difference in building orientation is important for judging whether buildings should be in the same building pattern. As shown in Figure 4, we derive the angle N°(N°∈[0,π]) between the width of the SBR (see Section 3.1.2.1) and the X axis. The expression for the descriptive variable is

(3) $$O=\{\begin{array}{c} \frac{90-N}{180},\;\;W>H,\\ \frac{180-N}{180},\;\;H>W \end{array}$$

where H and W are the height and width of the SBR, respectively, as defined in Section 3.1.2.1. The output value of the expression above is normalized during the calculation procedure to avoid the overfitting problem [33] during the training process.

#### 3.1.3. Graph Convolutional Network

The building group partition and building pattern recognition are based on the feature encoding of the building nodes in this study. The descriptive indices for buildings are given in Table 1. Similar to the human visual system, the GCN model makes judgments on the basis of the differences among the building and its Kth-order neighbors, as human’s eyes distinguish detail based on the gradient information of pixels.

As shown in Figure 3b, the shifting degree of adjacency weight (Section 3.1.2.1) is small when the building is located in the inner building group, while the shifting degree is relatively larger when the building is located on the edge of the building block (Figure 3a). This is one of the differences between the buildings in various states. In addition, differences are also embodied in the aspects of size, shape, orientation and other indices. The model learns the judgment rules by using the training samples.

**Figure 5:**
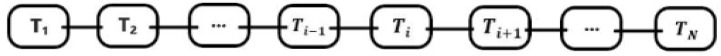
One-dimensional linear adjacency.

**Figure 6:**
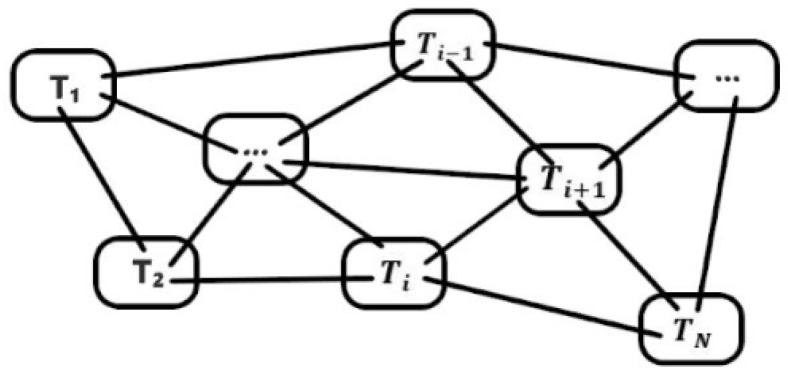
Sample of a real building distribution.

On the basis of the concepts above, the process for deriving building node encoding is as follows: First, we consider the situation of one-dimensional linear adjacency. As shown in Figure 5, only building $T_{i-1}$ and building $T_{i+1}$ are adjacent to building $T_{i}$. Therefore, the difference information $\delta_{i}$ is derived from the following aggregation operation:

(4) $$\delta_{i}=(\varphi_{i}-\varphi_{i+1})+(\varphi_{i}-\varphi_{i-1})$$

where $\varphi_{i}$ denotes the descriptive vector of building $T_{i}$. One sample of a real building distribution shown in Figure 6. Similar to Equation (4), the aggregation operation is given by

(5) $$\delta_{i}=\sum_{j=1}^{N}A_{i,j}\times \left(\varphi_{i}-\varphi_{j}\right)=\varphi_{i}\deg\left(i\right)-\sum_{j=1}^{N}A_{i,j}\varphi_{j}$$

where A refers to the adjacency matrix and deg(i) denotes $\sum_{j}A_{i,j}$.

For each building node in a graph, the computation process can be described based on the matrix operation

(6) $$\left[\begin{array}{c} \delta_{1}\\ \vdots \\ \delta_{N} \end{array}\right]=\left[\begin{array}{c} \deg\left(1\right)\varphi_{1}\\ \vdots \\ \deg\left(N\right)\varphi_{N} \end{array}\right]-A\left[\begin{array}{c} \varphi_{1}\\ \vdots \\ \varphi_{N} \end{array}\right]$$

We define vectors $\varphi ={\left[\varphi_{1},\varphi_{2},\ldots ,\varphi_{N}\right]}^{T}$ and $\delta =\left[\delta_{1},\ldots ,\delta_{N}\right]$ and get

(7) δ=Dφ−Aφ=(D−A)φ=Lφ

where L is the Laplacian matrix. Equation (7) shows that the usage of the Laplacian matrix is equivalent to the aggregation operation. A symmetric normalization operation for the Laplacian matrix [15] is implemented to address overfitting. The expression is

(8) $$L^{sys}=D^{-\frac{1}{2}}LD^{-\frac{1}{2}}=\;I_{N}-\;D^{-\frac{1}{2}}AD^{-\frac{1}{2}}$$

where $I_{N}$ is the identity matrix of size N.

The Fourier transform is an effective tool in the fields of signal analysis and image processing; it converts the original signal or image information into the Fourier domain [3,38]. In this study, we first extract the adjacency information from $L^{sys}$ (Equation (8)) by using the graph Fourier transform, and then we introduce a polynomial approximation operation implemented on the modified Laplacian matrix that optimizes the computational procedure.

First, the spectral decomposition for the Laplacian matrix is given by

(9) $$L=\;U\left[\begin{array}{ccc} \lambda_{1} & \cdots & 0\\ \vdots & \ddots & \vdots \\ 0 & \cdots & \lambda_{N} \end{array}\right]U^{-1}$$

where $U=\left(\vec{u_{1}},\;\vec{u_{2}},\;\ldots ,\;\vec{u_{N}}\right)$ and $\lambda_{n}\;\left(n\in \left[0,N-1\right]\right)$ is the *n*th eigenvalue of the Laplacian matrix. Because U is an orthogonal matrix, and thus $U^{T}=U^{-1}$, according to the definition of the Fourier transform, the graph Fourier transform [3] is

(10) $$F\left(\lambda_{l}\right)=\hat{f}\left(\lambda_{l}\right)=\sum_{i=1}^{N}\chi_{l}^{T}\left(i\right)f_{l}\left(i\right)$$

where $f_{l}$ refers to the signal (the input vector) and ${\left\{\chi_{l}\right\}}_{l=0}^{N-1}$ are the eigenvectors of the Laplacian matrix. The computing process in detail is

(11) $$\left[\begin{array}{c} \hat{f}\left(\lambda_{1}\right)\\ \vdots \\ \hat{f}\left(\lambda_{N}\right) \end{array}\right]=\left[\begin{array}{ccc} \chi_{1}\left(1\right) & \cdots & \chi_{1}\left(N\right)\\ \vdots & \ddots & \vdots \\ \chi_{N}\left(1\right) & \cdots & \chi_{N}\left(N\right) \end{array}\right]\;\left[\begin{array}{c} \vec{f_{1}}\\ \vdots \\ \vec{f_{N}} \end{array}\right]$$

The inverse Fourier transform is defined as $f_{l}=\sum_{i=0}^{N-1}\hat{f}\left(\lambda_{l}\right)\chi_{l}$. On the basis of the derivation above, the convolution can be first converted into a point-wise product in the Fourier domain, and then reconverted into the vertex domain [3] as follows:

(12) $$f*g=\sum_{l=0}^{N-1}\hat{f}\left(\lambda_{l}\right)\hat{g}\left(\lambda_{l}\right)\chi_{l}$$

In addition, in this study we introduce a polynomial approximation of the Laplacian matrix based on Chebyshev polynomials to obtain the following effects [3,32]:

- Aggregating the differences between each building and its Kth-order neighbors based on adjacency information;
- Realizing local weight sharing for the convolutional kernels, and;
- Reducing the computational cost for learning.

According to the recursion formula of Chebyshev polynomials, $T_{k}\left(X\right)=2XT_{k-1}\left(X\right)-T_{k-2}\left(X\right)$, where $T_{0}\left(X\right)=I_{N}\;\mathrm{and}\;T_{1}\left(X\right)=X$, we get the coefficients $\beta_{k}$. The approximation of the Laplacian matrix is designed as [32,34]

(13) $$L=U\left[\begin{array}{ccc} \sum_{k=0}^{K}\beta_{k}\lambda_{1}^{k} & \cdots & 0\\ \vdots & \ddots & \vdots \\ 0 & \cdots & \sum_{k=0}^{K}\beta_{K}\lambda_{n}^{K} \end{array}\right]U^{T}$$

The following derivation shows a clearer process:

(14) $$\begin{array}{c} L=\;\beta_{1}U\left[\begin{array}{ccc} \lambda_{1}^{1} & \cdots & 0\\ \vdots & \ddots & \vdots \\ 0 & \cdots & \lambda_{n}^{1} \end{array}\right]U^{T}+\ldots +\beta_{K}U\left[\begin{array}{ccc} \lambda_{1}^{K} & \cdots & 0\\ \vdots & \ddots & \vdots \\ 0 & \cdots & \lambda_{n}^{K} \end{array}\right]U^{T}=\beta_{1}{\left(U\left[\begin{array}{ccc} \lambda_{1}^{1} & \cdots & 0\\ \vdots & \ddots & \vdots \\ 0 & \cdots & \lambda_{n}^{1} \end{array}\right]U^{T}\right)}^{1}+\\ \ldots +\beta_{K}{\left(U\left[\begin{array}{ccc} \lambda_{1}^{1} & \cdots & 0\\ \vdots & \ddots & \vdots \\ 0 & \cdots & \lambda_{n}^{1} \end{array}\right]U^{T}\right)}^{K}=\beta_{1}L^{1}+\ldots +\beta_{K}L^{K}=\sum_{j=1}^{K}\beta_{j}L^{j} \end{array}$$

**Figure 7:**
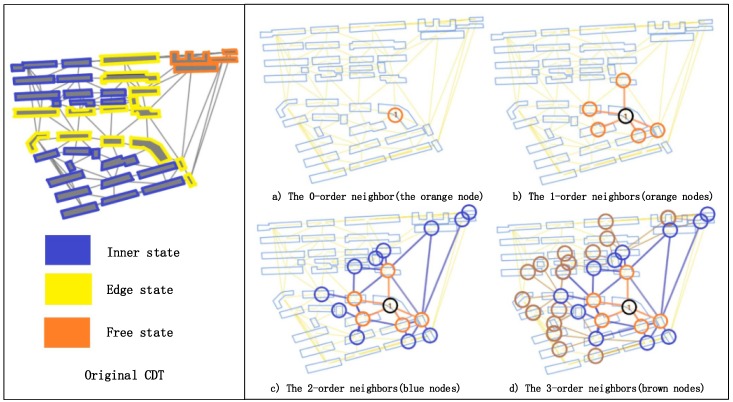
Visualization of the perception with different polynomial orders K. (**a**) The 0-order neighbor (the orange node). (**b**) The 1-order neighbors (orange nodes). (**c**) The 2-order neighbors (blue nodes). (**d**) The 3-order neighbors (brown nodes).

Equation (14) shows that calculating the eigenvectors is not required, which simplifies the computational procedure. To match the requirement that the range of the input eigenvalues is [−1,1], the following transform is operated on the Laplacian matrix before inputting:

(15) $$\tilde{L}=\frac{2}{\lambda_{max}}L-I_{N}$$

where $\lambda_{max}$ is the maximum of the eigenvalues. In terms of the Laplacian matrix L derived from a graph G, only the coordinate values referring to two adjacent buildings are 1, whereas the others are 0. Therefore, only same-order neighbors share the same weight from the same convolution kernel according to Equation (14), and the property of local weight sharing for kernels is realized, which also enlarges the perception of adjacent regions of the starting building node (Figure 7) for the GCN model with the settable parameter K shown in Equation (14).

Based on the derivation above, the graph convolution formula is given by f∗g=(Lφ)∗g. After making the low-order polynomial approximation for the Laplacian matrix (Equation (14)), we get the size of the output matrix as (N,Kn), where N is the number of the vertices in a graph and n denotes the length of the descriptive vector of buildings. Therefore, the size of the convolution kernels is (Kn,I), where I refers to the number of convolution kernels. In general, the size of kernels is proportional to the product of K and n, which demonstrates that the computing cost is minimized compared with the classical graph convolution method [36].

Training of the GCN model is based on the gradient descent method to minimize the output loss of the model. According to the chain rule, the gradient expressions used in the back-propagation process [39] are as follows:

(16) $${\left(\frac{бloss}{бw_{i.j}^{\left(k\right)}}\right)}^{\left(l\right)}=x_{i}^{\left(k\right)}\delta_{j}^{\left(l+1\right)},\;i\in \left[0,n_{f}-1\right],\;j\in \left[0,n_{k}-1\right],\;k\in \left[0,K-1\right]$$

(17) $$\delta_{i}^{\left(k,l\right)}=\sum_{j=0}^{n_{k}-1}w_{i,j}^{\left(k\right)}\delta_{j}^{\left(l+1\right)}$$

where $n_{f}$ denotes the length of the input vectors and $n_{k}$ refers to the number of convolution kernels.

**Figure 8:**
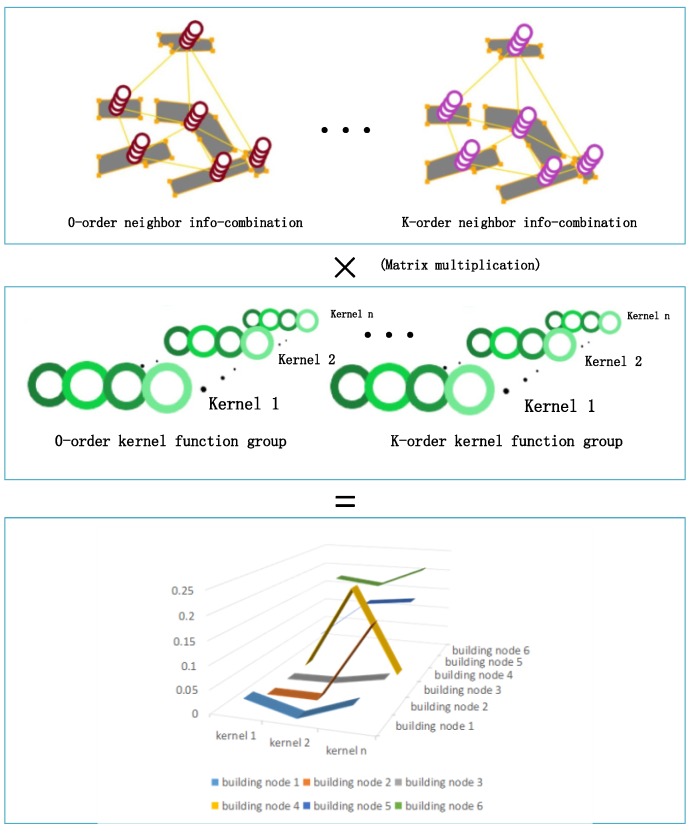
Visualization of the computing process for the graph convolution operation.

In this study, the GCN model consists of two convolutional layers. The graph convolution operation is shown in Figure 8.

There are three kinds of building states, thus there are three convolution kernels in the second layer, and Softmax is the selected as the activation function. Each component of the output vector denotes the probability of the related class.
**Algorithm 1** BFS (G, $V_{s}$, v, $S_{n1}$)1:  Initialize: $S_{t}$ (an empty stack for the BFS algorithm). 2:  push v to $S_{t}$ 3:  while not empty ($S_{t}$) do 4:      v ← $S_{t}.pop()$ 5:     $l_{v}$ = [neighbors (v)] 6:     for each $v_{j}\;\in l_{v}$ do 7:        if $v_{j}$’s state is $S_{n}$ then 8:            append $v_{j}$ to $V_{s}$ 9:            push $v_{j}$ to $S_{t}$ 10:  Return: $V_{s}$

**Algorithm 2** Inner state node searching process for group pattern reconstruction1:  Input: Building graph *G* = (*V*, *E*) (where *V*[*i*] stores the state of building i recognized by the GCN (Section 3.1.3)); number of building nodes = *N* 2:  Initialize: $V_{Inner}$ (an empty list to store the building groups of inner state), $N_{Inner}$ = 0 3:  for i = 0 to *N* − 1 do 4:     if *V*[*i*] is Inner state and have not been appended to $V_{Inner}$ then 5:       $N_{Inner}++$, append(V[i]) to $V_{Inner}[N_{Inner}]$ 6:       *BFS* (*G*, $V_{Inner}[N_{Inner}]$, V[i], Inner state) ◁*BFS*(Algorithm 1) for node traversing 7:  for *i* = 0 to *N* − 1 do 8:     if *V*[*i*] is edge state and have not been appended to $V_{Inner}$ then 9:        for *j* = 0 to $N_{Inner}$ − 1 do 10:          for *k* = 0 to $V_{Inner}\left[j\right].length$ − 1 do 11:            if V[i] $\in \mathrm{neighbors}\;(V_{Inner}\left[j\right]\left[k\right])$ and $V_{Inner}\left[j\right]\left[k\right]$ is Inner state then 12:               append(V[i]) to $V_{Inner}\left[j\right]\left[k\right]$ 13:  Return: $V_{Inner}$

**Algorithm 3** Edge state node searching process for group pattern reconstruction1:  Input:  Building graph *G* = (*V*, *E*) (where *V*[*i*] stores the state of building i recognized by the GCN (Section 3.1.3)); number of building nodes = *N* 2:  Initialize: $V_{edge}$ (an empty list to store building groups of edge state), $N_{edge}$ = 0 3:  for i = 0 to *N* − 1 do 4:     if *V*[*i*] is edge state and have not been appended to $V_{Inner}$ or $V_{edge}$ then 5:       $N_{edge}++$, append(V[i]) to $V_{edge}[N_{edge}]$ 6:       *BFS* (G, $V_{edge}[N_{edge}]$, *V*[*i*], Edge state) ◁*BFS*(Algorithm 1) for node traversing 7:  Return: $V_{edge}$

**Algorithm 4** Free state node searching process for group pattern reconstruction1:  Input: Building graph *G* = (*V*, *E*) (where *V*[*i*] stores the state of building i recognized by the GCN (Section 3.1.3)); number of building nodes = *N* 2:  Initialize: $V_{free}$ (an empty list to store the building groups of free state), $N_{free}$ = 0 3:  for i = 0 to *N* − 1 do 4:     if V[i] is free state and have not been appended to $V_{free}$ then 5:        $N_{free}++$, append(V[i]) to $V_{free}[N_{free}]$ 6:        *BFS* (G, $V_{free}[N_{free}]$, V[i], free state) ◁*BFS*(Algorithm 1) for node traversing 7:  Return: $V_{free}$


### 3.2. Building Group Partition Algorithm

**Figure 9:**
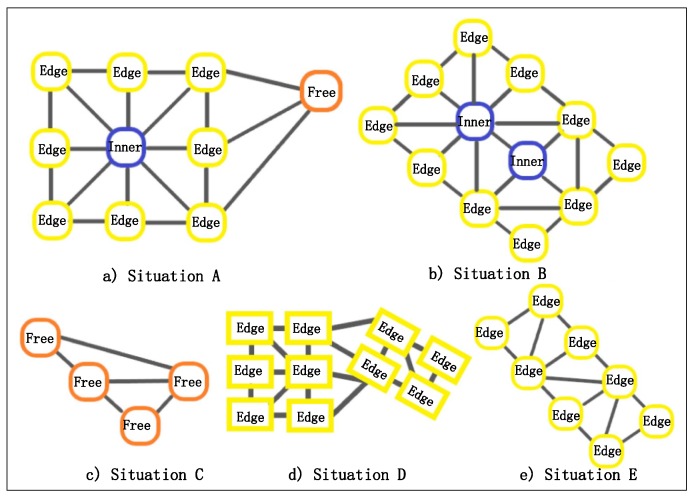
Clustering samples of different state building nodes. (**a**) Situation A. (**b**) Situation B. (**c**) Situation C. (**d**) Situation D. (**e**) Situation E.

As was stated in Section 3.1, the building nodes in a graph have been classified into three types: edge state nodes, inner state nodes and free state nodes. Through the distribution of buildings is extremely random, after the classification of building states, finite kinds of situations are revealed as follows:

- Situations of inner state: There is only one existing situation of inner state nodes in a building block. As shown in Figure 9b, the inner state nodes are surrounded by the edge state nodes, and form the building block with the latter. Algorithm 2 is used for obtaining the building block containing inner state nodes.
- Situations of edge state nodes: As shown in Figure 9b,e there are two possible situations of edge state nodes: 1. forming the building block with inner state nodes and 2. forming the building block only consisting of edge state nodes. Algorithm 2 is adapted to the first situation, while Algorithm 3 is applied to the second situation.
- Situations of free state nodes: There is only one possible situation of free state nodes because of its independence compared to the other two kinds of building nodes, as shown in Figure 9c. Algorithm 4 is used for constructing the building group consisting only of free state nodes.
- Building nodes in the same state, but not in the same building block: It is possible that, though the nodes are identified as nodes in the same state, they belong to different building blocks (Figure 9d). Moreover, a fine-grained partition is needed because of the differences in the aspects of size, outline and orientation because the group partition step mainly focuses on the spatial distribution. This problem will be solved in the following step.

**Algorithm 5** Building node clustering1:   Input:  Building graph *G* = (*V*, *E*) (where $V_{i}$ ∈ *V* means the building nodes from the same building division (Section 3.2)); *N* means the number of buildings 2:  Initialize:  $S_{l}$ (an empty list to store building pattern groups), *M* (a list initialized by False, storing the state if $V_{i}$ have been checked), $S_{t}$ (an empty stack for the BFS algorithm), RF (a function using the RF model to judge whether the buildings should be in the same pattern; see Section 3.3), *L* = *N*, n = −1 3:  while *L* > 0 do 4:     *L* --, n ++, *T* ← None 5:     append ($V_{i}$) to $S_{l}\left[n\right]$, push $V_{i}$ to $S_{t}$          ◁ where $V_{i}\in$ V and M[i] == False 6:       while not empty ($S_{t}$) do 7:        $V_{i}$ ← $S_{t}.pop()$, M [i] ← True 8:        $l_{v}$ = [neighbors ($V_{i}$)] 9:        sort $\left(l_{v}\right)$             ◁ by the distances to $V_{i}$ in ascending order 10:      for each $V_{j}\;\in l_{v}$ do 11:          if *T* == None and *M*[ i] == False then 12:             if RF $(V_{i}$, $V_{j})$ is True then 13:                *T* ← $V_{j}$, append $\left(V_{j}\right)$ to $S_{l}\left[n\right]$, push $V_{j}$ to $S_{t}$, *L* -- 14:          else if T!=None and M [i] == False then 15:             if RF $(V_{i}$, $V_{j}$, T) is True then 16:                append $\left(V_{j}\right)$ to $S_{l}\left[n\right]$, push $V_{j}$ to $S_{t}$, *L* -- 17:  Return: $S_{l}$

### 3.3. Fine-Grained Partition for Building Blocks

As was stated in Section 3.2, the building group partition step divides building nodes into building blocks according to the spatial distribution. In this study, we use a fine-grained partition method to extract building patterns based on the similarity of the buildings.

**Figure 10:**
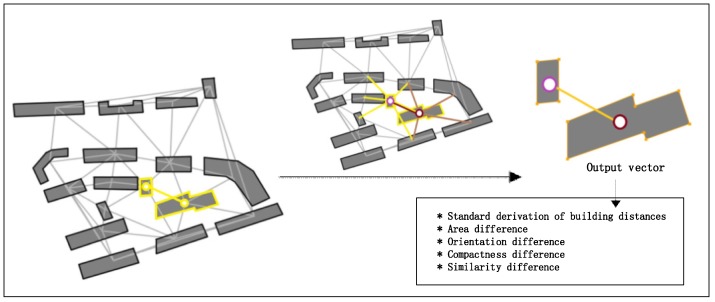
Visualization of the descriptive indices of the difference between building node pairs.

We employ indices of the standard deviation (SD) of these building distances [4], the area difference, the orientation difference, the compactness difference and the similarity difference [3], to construct the discriminant vector (Figure 10). The five indices quantify the difference in the related building nodes. In this study, the RF model [4] is utilized to judge whether the buildings are in the same pattern. Algorithm 5 is used for building node clustering to accomplish the fine-grained partition.

### 3.4. Building Pattern Recognition

The GCNN model used for building pattern recognition consists of graph convolutional layers and a deep neural network. In this part, we first derive the adjacency information from the coordinate data of building contours through the CDT method. The descriptive vectors are constructed by the shifting degree of the adjacency weight (Section 3.1.2.1) of the key points from the contours. The input matrix is constructed from the descriptive vectors of the key points in a building pattern, and the adjacency matrix recording the adjacency information so that the model only focuses on the topological relationships among the buildings.

**Figure 11:**
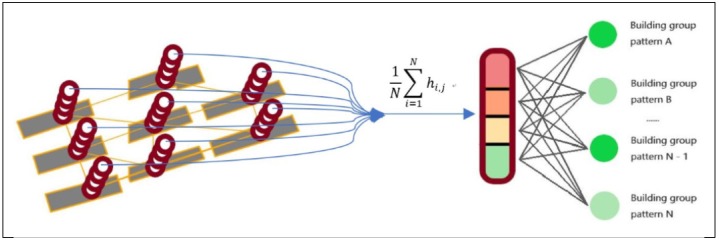
Visualization of the method of connecting the convolutional layer and the fully connected layer.

The connection of the GCN to the deep neural network is shown as Figure 11. The size of the output matrix of the graph convolutional layer is (N,Kn) (Section 3.1.3), where N is the number of the vertices in a graph and n denotes the length of the descriptive vector of the nodes. The representation vector of a graph $G_{i}$ is derived by using [40]

(18) $$h_{g}=\frac{1}{N_{vertex}}\sum_{i=1}^{N_{vertex}}h_{v}$$

where $h_{v}$ denotes the output of the graph convolutional layer and $h_{g}$ is the input of the fully connected layer. The training method is the same as mentioned in Section 3.1.3.

## 4. Framework for Building Pattern Recognition

**Figure 12:**
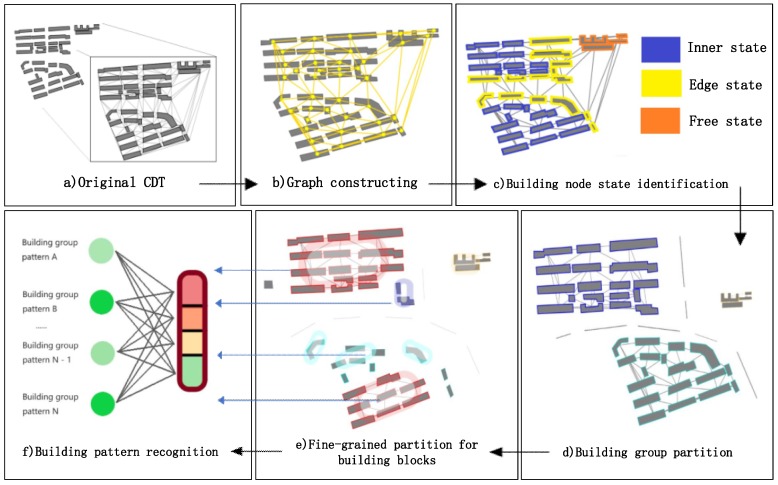
Description of building group pattern abstraction and detection algorithm framework. (**a**) Original CDT. (**b**) Graph constructing. (**c**) Building node state identification. (**d**) Building group partition. (**e**) Fine-grained partition for building blocks. (**f**) Building pattern recognition.

In general, based on the methods described in Section 3, to achieve building pattern recognition, the algorithm framework proposed in this study consists of the following five parts:

- Graph construction for buildings. Each building node has its own unique identification number. As shown in Figure 12a,b we first derive the adjacency information through constructing a constrained Delaunay triangulation (CDT) [4,41,42] for all the buildings.
- Building node state identification. Based on the vector data of building contours, each building entity is described by indices including its area, perimeter, orientation, compactness and shifting degree of adjacency weight (Section 3.1.2.1), and then a descriptive vector is constructed. The descriptive vectors are the input to the GCN model in the subsequent step. To make rules for building group partition, three states (edge state, inner state and free state) (Section 3.1.1) are defined to describe the spatial state of the buildings. The related dataset of building node state labeling is constructed, and the GCN model with semi-supervised learning is trained to enhance the ability of generalization. The GCN model is used to identify the building node state, which is indispensable for the building group partition algorithm (see Section 3.2). The partition process and the building samples identified as different states are shown in Figure 12c.
- Building group partitioning. The building group partition algorithm based on the identification results of the building state is run. The outputs of the algorithm are the building blocks from the partitioned building groups (Figure 12d).
- Building node clustering. A breadth-first search (BFS) is used to traverse building nodes in a graph, and the graph of each building block is constructed by CDT. A random forest (RF) algorithm is introduced to judge whether two or three building entities (Section 3.3) can be categorized into the same building pattern. The objective of this step is to extract all the separate building patterns in a building block (Figure 12e).
- Building pattern recognition. In this final step, a GCNN model (Section 3.4) is used to recognize the building patterns, as shown in Figure 12f. The model is trained with supervised learning with the building node pattern datasets.

To make a more intuitive description, the specific process of the framework is summarized in Algorithm 6, and the whole workflow is shown in Figure 12.
**Algorithm 6** Framework for building pattern recognition1:   **Require:** 2:    *X*: denotes the data of a building block selected in advance. 3:    *X_j_*: denotes primary data of a building object with ID *j*, including the coordinate data of 4:       the building contours. 5:  **Step 1:** Construct a graph *G* for *X* using CDT. 6:  **Step 2:** Calculate the values of the variables mentioned in Section 3.1.2 to construct a 7:  new descriptive vector for each building object $X_{j}$, on the basis of the adjacent relations 8:  derived from the graph *G*. 9:  **Step 3:** Classify each building *X_j_* into the state *S_j_* by the GCN model (see Section 3.1.3). 10:  **Step 4:** Accomplish building graph partition. Functions $f_{a2}$, $f_{a3}$ and 11:  $f_{a4}$ stand for Algorithms 2–4 respectively. 12:    $V_{Inner}$ = $f_{a2}\left(G\right)$ 13:    $V_{edge}$ = $f_{a3}\left(G\right)$ 14:     $V_{free}=f_{a4}\left(G\right)$ 15:  Where $V_{Inner}$[*i*] denotes the $i^{th}$ building group and $V_{Inner}$ [*i*] = [ $X_{0}$ … $X_{k-1}$]. k stands for 16:  the number of the buildings of the $i^{th}$ building group. Data structures of $V_{edge}$ and 17:  $V_{free}$ are the same as $V_{Inner}$. 18:  **Step 5:** Utilize the RF model to accomplish the fine-grained partition (Section 3.3) for 19:  the building groups from $V_{Inner}$, $V_{edge}$ and $V_{free}$. 20:  **Initialize:**
21:    *S_l_*: an empty list. 22:    V: a list consists of all the building groups from $V_{Inner}$, $V_{edge}$ and $V_{free}$. 22:  **for**
*i* = 0 to V.length − 1 do 23:    $G^{\prime }$ = V [*i*] 24:    $S^{\prime }$ = $f_{a5}\;\left(G^{\prime }\right)$             ◁ $f_{a5}$ stands for the function of Algorithm 5 25:    **for** j = 0 to $S^{\prime }.length$ − 1 do 26:      append $S^{\prime }\left[j\right]$ to $S_{l}$     ◁ $S^{\prime }\left[j\right]$ denotes a building group 27:    **end for** 28:  **end for** 29:   **Step 6:** Classify the building group into the pattern with the GCNN model. 30:  **for**
*i* = 0 to $S_{l}.length$ − 1 do 31:    $p^{\prime }$ = $GCNN\left(S_{l}\left[i\right]\right)$         ◁ $p^{\prime }$ means the classifying result 32:  **end for**


## 5. Experiments and Results

### 5.1. Building Node State Recognition

**Figure 13:**
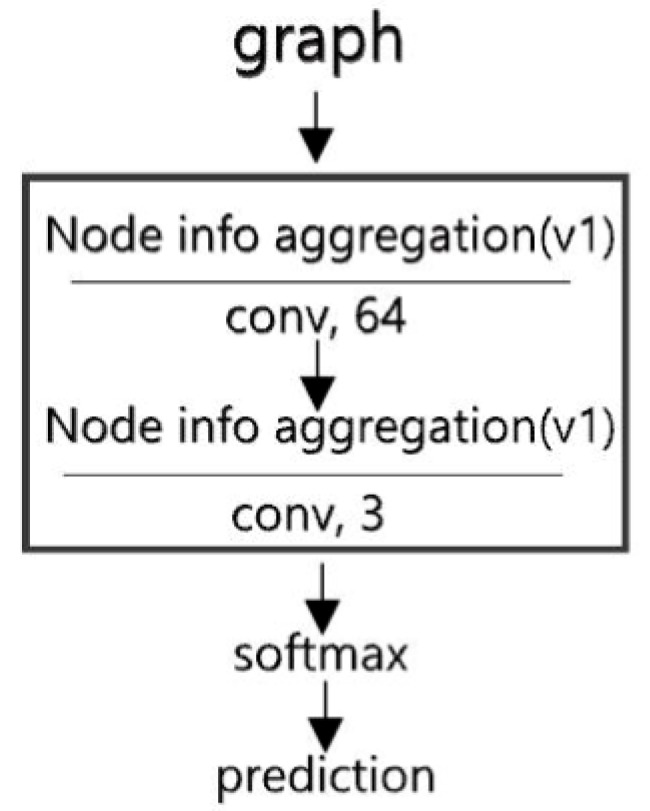
Structure of the graph convolutional network (GCN) model for building node state identification. Node info aggregation(v1) refers to using the low-order polynomial approximation method (Section 3.4).

In the task of building state identification (Section 3.1.3), the output of the GCN model is a probability vector ${\left\{P_{i}\right\}}_{i=0}^{M-1}$, where M denotes the number of possible classes. If $P_{i}$ is the maximum, then i refers to the class in which the object belongs. As was stated in Section 4, the graph generated by CDT is the input of the GCN model. The model structure, shown in Figure 13, consists of two convolutional layers with 64 kernels and 3 kernels, respectively. The Softmax function is selected as the activation function of the last layer, and its output is a probability vector. During the training process, we use the Adam optimization algorithm as the optimizer. The regularization weight is set as $5\times 10^{-4}$. To enhance the generalization ability, the model is trained with semi-supervised learning [15], and the dropout probability is set as 0.5. Meanwhile, the model is trained by one graph per step.

**Figure 14:**
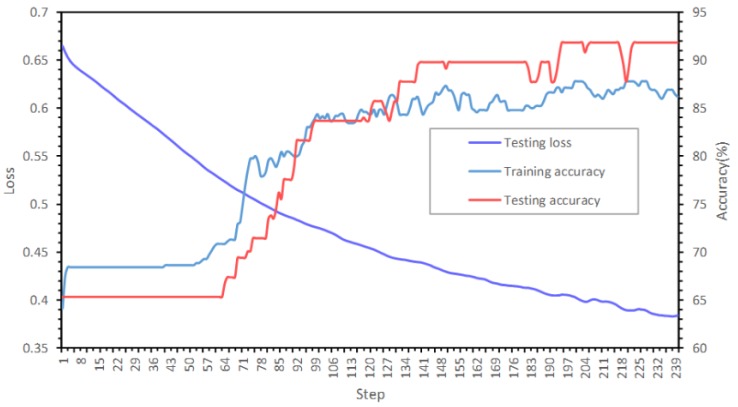
Training and testing results from the GCN model for building node state identification.

As shown in Figure 14, after training on the Beijing Xicheng District dataset for 40 epochs, the training accuracy achieved 86.05% with the semi-supervised learning algorithm. The testing accuracy on the Xi’an dataset achieved 92.71%, and the testing loss was 0.362.

**Figure 15:**
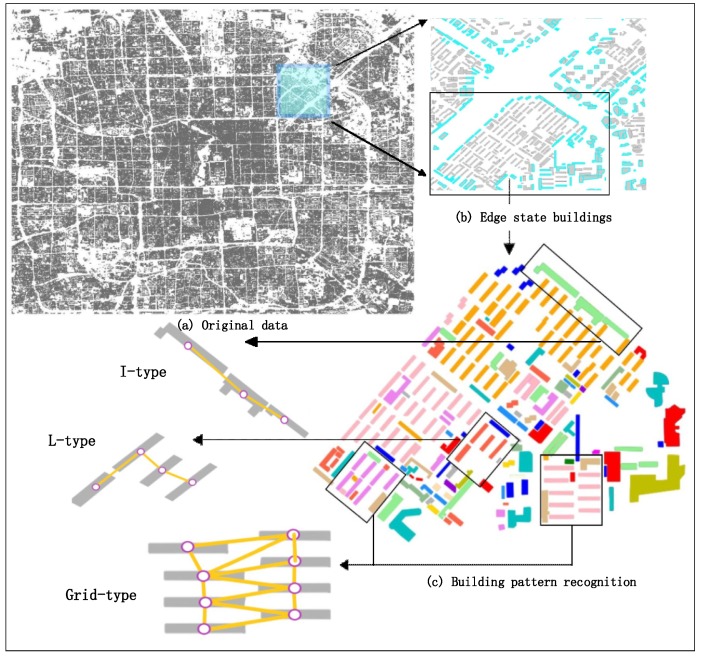
Partial results from the proposed algorithm framework.

The confusion matrix is shown in Table 2, and the kappa coefficient is 0.832. Given that the training set and the testing are based on data from two typical cities in China, the accuracies and the two curves indicate the good generalization ability of the GCN model. Figure 15b shows the partial results of the trained model on the testing data. The buildings with blue contours are identified as edge state buildings.

**Figure 16:**
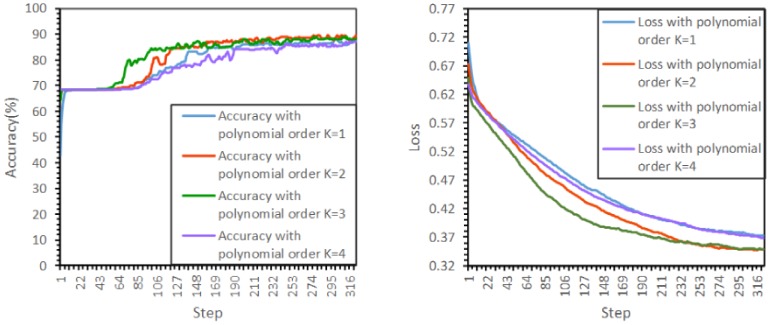
Test results from the GCN model for building node state identification with different polynomial orders K.

Figure 16 shows the training accuracies and losses with different polynomial orders K. The results indicate that the performance is poor when K=1, but performance begins to improve from K=2 to K=3. When K=4, the accuracy decreases. This confirms that a larger perception region helps improve the accuracy. In addition, the findings indicate that the best performance emerges when K=3, with higher values of K having an adverse impact.

### 5.2. Fine-Grained Partition

The RF model is employed to judge whether the buildings belong to the same building pattern for the fine-grained partition task (Section 3.3). The Beijing Xicheng District dataset is used for model training, while the Xi’an dataset is used for testing. The input vectors are prepared by following the steps described in Section 3.3, and the RF model is trained with supervised learning. In addition, a support vector machine (SVM) model is trained under the same conditions for comparison [3]. The experimental results, given in Table 3, indicate the RF method achieves better performance. The experiment confirms that the effect of the proposed method is satisfactory, and the generalization ability of the RF model is strong enough to be applied to other datasets.

### 5.3. Building Group Pattern Recognition and Comparative Analysis

**Figure 17:**
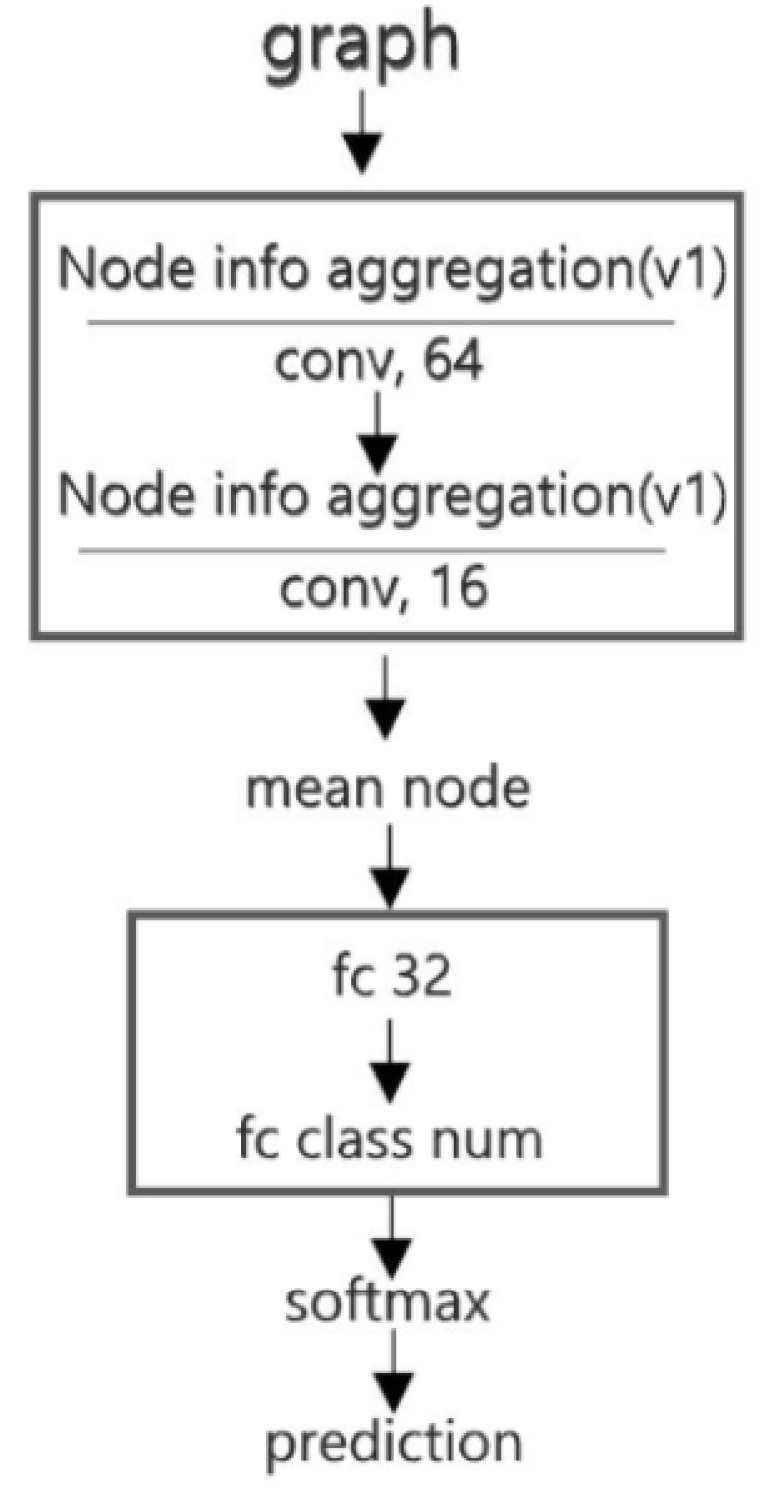
Structure of the graph convolutional neural network (GCNN) model. Node info aggregation(v1) refers to using the low-order polynomial approximation method (Section 3.1.3).

**Figure 18:**
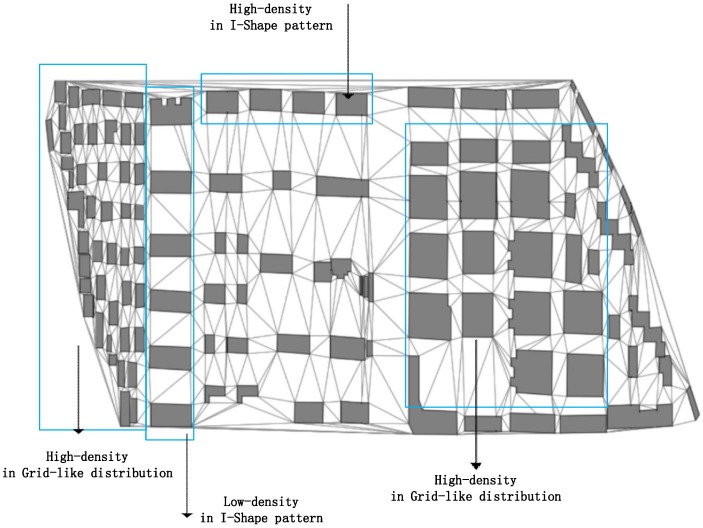
Part of the pattern recognition results with different densities.

The structure of the GCNN model used for the building pattern recognition task is shown in Figure 17. In this experiment, the Beijing Xicheng District dataset is used for model training and the Xi’an dataset is used for testing. Table 4 shows the results: the accuracies of training and testing are 98.20% and 89.83%, respectively. In addition, the confusion matrix of the recognition result on the testing set (Xi’an dataset) is shown in Table 5. The kappa coefficient is 0.847. Two samples of recognition results on the testing set are shown in Figure 15c and Figure 18.

The following comparative experiment was done for better testing the advantages and disadvantages of the proposed method. Indices of mean distance [4], standard deviation of building distances [4], black-and-white ratio [43] and area ratio of the building to the smallest bounding rectangle of the group [4] are employed to construct the descriptive vectors for the building patterns. The SVM model and the RF model were utilized for comparison, and the descriptive vectors of the building patterns extracted from the datasets were used as the input of the models. The comparative results are listed in Table 4. Both the SVM method and the RF method have high training accuracy, but their testing accuracy is not good. The results can be explained by the fact that the descriptive vector input to the SVM model and the RF model leads to the sensitivity to spatial distribution of the buildings in various geographical regions, while the random distribution of the buildings in the datasets leads to the density being extremely not stationary. As shown in Figure 18, the building patterns that are the same class but have different density of buildings cause difficulties for the models in learning generalization features and identification rules. By contrast, the GCNN model in this study only focuses on the adjacency relationship.

Therefore, the proposed method is not spatial distribution sensitive, especially when the training data and testing data are not from the same dataset.

As in the experiment above, five indices (standard deviation of building distances, similarity difference, area difference, orientation difference and compactness difference among the building and its neighbors) [3] are utilized to construct the descriptive vector for one building node. The vectors are used as the input to the SVM model and the RF model. The two models are trained by using the same datasets as for the GCN model (Section 5.1). Table 6 lists the comparative results, which indicate that the accuracy of the GCN method is the best on the testing dataset, while the generalization ability of the other two methods is relatively poor.

### 5.4. Parameter Descriptive Ability Analysis

**Figure 19:**
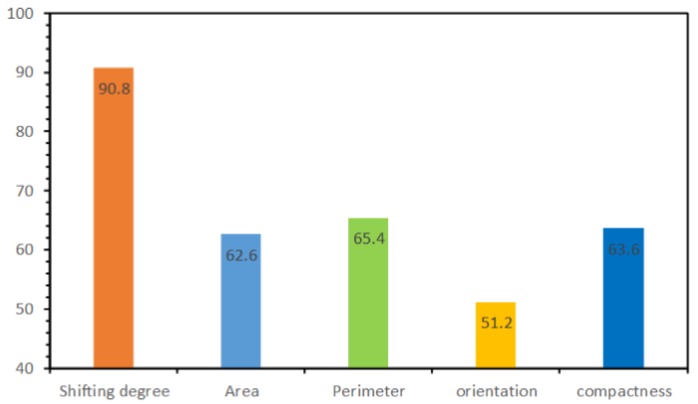
Experimental results for the descriptive abilities of different indices.

In this experiment, we examined the descriptive ability of each descriptive variable. We used a single variable to construct the input vectors and repeated the experimental steps of building node state identification. The results shown in Figure 19 indicate that the testing accuracy (90.8%) is close to the accuracy (92.71%) of the original experiment (Section 5.1) when the input vectors are constructed only by the shifting degree of adjacency weight (Section 3.1.2.1). Therefore, it can be inferred that, for the task of building group partition that is related to human visual perception, the descriptive ability of the shifting degree of adjacency weight is effective. In addition, the findings also confirm that the concept of using the shifting degree of adjacency weight is reasonable in machine-learning tasks based on topological graphs when relating to visual perception.

### 5.5. Model Structure Exploration Results

**Figure 20:**
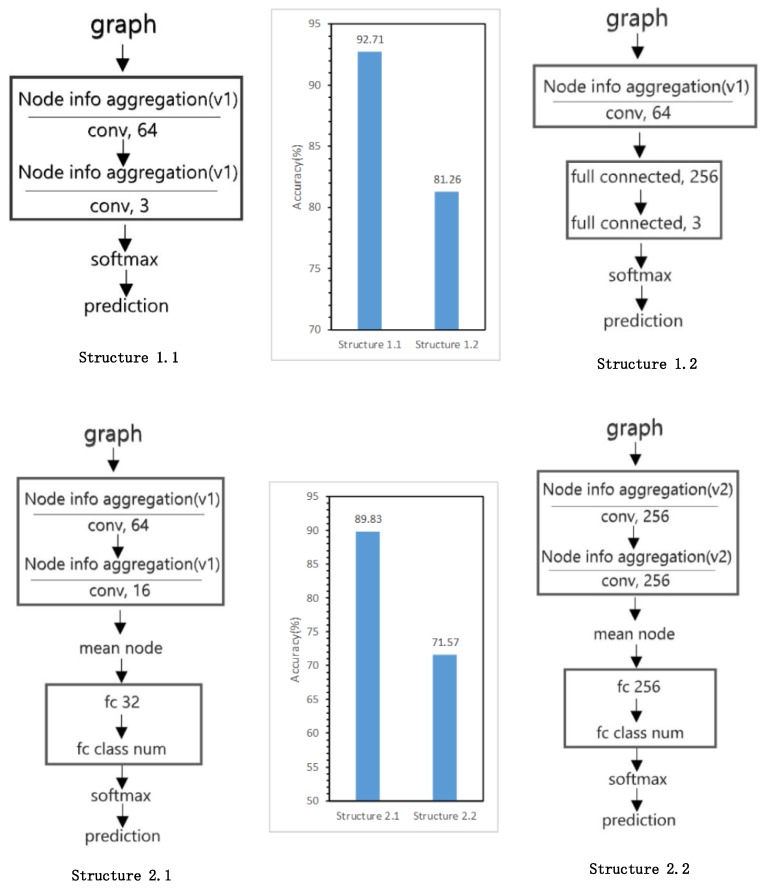
Experimental results for network structure exploration.

Different strategies of model structure have different applicability in the tasks of building state identification and building pattern recognition. This experiment is aimed to explore the difference of the performances with different model structures. Figure 20 shows the model structures and the related experimental results using the Xi’an dataset for testing.

Figure 20 (Structures 1.1 and 1.2) show that the classification ability of the fully connected layer is not good compared with the graph convolutional layer in the building state identification task, and the total precisions are 81.26% and 92.71%, respectively. Figure 20 (Structures 2.1 and 2.2) show that, in the task of building pattern recognition, using the convolution operation described in Section 3.1.3 offers better performance than the classical graph convolution method [36], as the accuracies on the testing set are 89.83% and 71.57%, respectively, while the latter requires a greater amount of computation.

## 6. Discussion

### 6.1. Spatial Adaptive Algorithm Framework Using GCNs

The GCN model and the GCNN model is not spatial distribution sensitive in the building pattern recognition task, because they only focus on the adjacency information and the characteristic differences between the building and its neighbors. By comparison, the RF-based method [4] is most likely limited because the variables input measure the indices in Euclidean space directly which lead to a bad performance, since some spatial features, such as the sparsity of the building blocks, sizes and the geometries of the buildings are very flexible and various. The experiments have demonstrated that our method for building pattern recognition outperforms the existing related methods, especially from the aspects of generalization ability and testing accuracy.

In addition, by combining the GCN model and the proposed algorithms, this bottom-up method can perceive the relationships among the building and its neighbors in the process of building a group partition, without the requirement of ancillary data (e.g., road networks and rivers).

### 6.2. Remaining Issues

As seen in Section 5, though the GCN model and the GCNN model are better in terms of generalization ability and testing results, their training accuracies are not satisfactory. One of the reasons for this is the existence of ambiguous situations during the annotation process.

**Figure 21:**
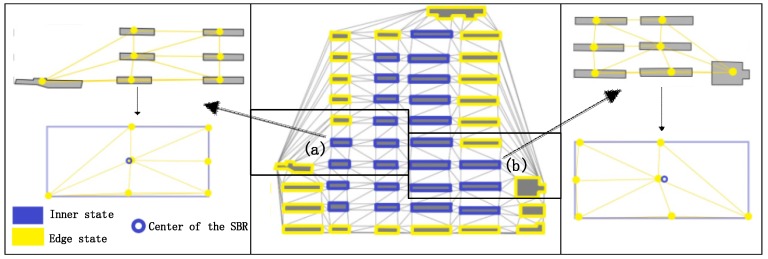
Recognition results from the GCN model for building state identification.

A sample is shown in Figure 21. Intuitively, buildings (a) and (b) should be in the edge state (Section 3.1.1), given their position in the building block. However, given the adjacency information and the small shifting degree of adjacency weight (Section 3.1.2.1), these buildings can reasonable be identified as inner state buildings. Such ambiguous situations lead to difficulty in making a precise graph dataset, destabilizing training and decreasing accuracy.

In addition, CDT is constructed for the coordinate data of the building contours as the input to the GCNN model (Section 3.4). However, another method of constructing CDT based on the center points of the building nodes [3] is not employed in this study, because the performance is often poor when the GCNN model is used in small graphs.

Therefore, an obvious limitation of the proposed method is that the topological structures of the same building patterns are not always stationary because of the various shapes of the buildings, and this leads to difficulty for the learning of the model.

## 7. Conclusions and Future Works

### 7.1. Conclusions

In this research, an algorithm framework for building pattern extraction and recognition combining the graph convolution operation, the RF model, a neural network and spatial adaptive algorithms, has been proposed. Besides, the multi-stage design of the framework is to achieve building pattern extraction which is associated with the multi-object detection task on topological data. The shifting degree of adjacency weight proposed in this research is utilized in order to exploit the distribution features of the building nodes and spatial adjacent relations. Experiments confirm the effectiveness of the descriptive vector constructed by the shifting degree of adjacency weight and other variables (e.g., the area, perimeter, orientation and compactness). Additionally, training and testing results indicate good generalization ability of the GCN model, since the training set and testing set are derived from the two various regions, Beijing’s Xicheng District and the core areas of the city of Xi’an, which shows that the proposed method is not spatial distribution sensitive. Another superiority is that this framework enables the building group partition task to be performed without any ancillary data. In addition, our study confirms the feasibility of using the graph convolution method to address the problem of building pattern recognition through a sample experimental study.

### 7.2. Future Works

We explored the applicability of different model structures and derived an ideal effect with reasonable computation cost. In the future works, the improvement of the aggregation operation (Section 3.1.3) is one of the important options for increasing the accuracy, since we simply calculate the gradients of the vectors in the research. More effort will be put into devising better descriptive methods for buildings and exploring more powerful models. In addition, as is stated in Section 6.2, the performance degradation of the GCNN model in small graphs is worthy of research in the future. Last but not least, solving the problem of ambiguous situations during the annotation process is essential, otherwise the performance of the models will be hard to be improved for the lack of precise graph datasets.

## Figures and Tables

**Table 1:** Description of spatial characteristics of individual buildings along with their equations and short descriptions.

Variable	Index	Equation	Description
Position feature	Shifting degree of adjacency weight in width direction	-	See Section 3.1.2.1
	Shifting degree of adjacency weight in height direction	-	See Section 3.1.2.1
Size	Area index	$A_{b}/\max(A_{b})$	Building area with normalizing operation Building perimeter with normalizing operation
	Perimeter index	$P_{b}/\max(P_{b})$	
Orientation	Orientation index	-	See Section 3.1.2.2
Shape	Compactness	$\frac{4\pi A_{b}}{P_{b}^{2}}$	Quadratic relationship between the area and the perimeter [37]
	Concavity	$\frac{A_{b}}{A_{ch}}$	Area ratio of the building to its convex hull [37]

**Table 2:** Confusion matrix of the building node state identification experiment with the dataset of the core areas of Xi’an.

Number of Examples = 950	Actual Inner State	Actual Edge State	Actual Free State
Predicted inner state	668	24	1
Predicted edge state	37	184	2
Predicted free state	0	0	34

**Table 3:** Comparative results of the two methods for the building node clustering task.

Method	Training Accuracy (Beijing Xicheng District)	Testing Accuracy (Xi’an)
SVM	98.30%	84.35%
RF	99.06%	96.77%

**Table 4:** Comparative results of the three methods for the building pattern recognition task.

Method	Training Accuracy (Beijing Xicheng District)	Testing Accuracy (Xi’an)
SVM	99.68%	77.18%
RF	99.45%	81.78%
GCNN	98.20%	89.83%

**Table 5:** Confusion matrix of the building pattern recognition experiment with the dataset of the core areas of Xi’an.

Number of Examples = 354	Actual I-Shape	Actual L-Shape	Actual Grid-Like
Predicted I-shape	118	9	11
Predicted L-shape	6	109	7
Predicted Grid-like	1	2	91

**Table 6:** Comparative results of the three methods for the building node state identification task.

Method	Training Accuracy (Beijing Xicheng District)	Testing Accuracy (Xi’an)
SVM	94.52%	81.35%
RF	96.84%	89.39%
GCNN	86.05%	92.71%
